# Surgical and Patient-Reported Outcomes of Delayed Anastomotic Urethroplasty for Male Pelvic Fracture Urethral Injury at a Japanese Referral Center

**DOI:** 10.3390/jcm11051225

**Published:** 2022-02-24

**Authors:** Akio Horiguchi, Masayuki Shinchi, Kenichiro Ojima, Yusuke Hirano, Keiichi Ito, Ryuichi Azuma

**Affiliations:** 1 Department of Urology, National Defense Medical College, Saitama 359-8513, Japan; shinchimasayuki@gmail.com (M.S.); dr.ojimax.navy@gmail.com (K.O.); yuusuke3558@gmail.com (Y.H.); itok@ndmc.ac.jp (K.I.); 2 Department of Plastic Surgery, National Defense Medical College, Saitama 359-8513, Japan; azuma@ndmc.ac.jp

**Keywords:** pelvic trauma, urethral trauma, erectile function, urinary continence, urethral reconstruction, patient-reported outcome

## Abstract

We aimed to assess the surgical and patient-reported outcomes of delayed anastomotic urethroplasty (DAU) for pelvic fracture urethral injury (PFUI). We included 211 male patients who underwent DAU for PFUI. DAU success was considered when the urethral lumen was sufficiently large for the passage of a flexible cystoscope, without additional treatment required. The patients completed the lower urinary tract symptoms (LUTS)-related quality of life (QOL) questionnaire (scores: 0, not at all; 1, a little; 2, somewhat; 3, a lot), EuroQol-5 dimensions (EQ-5D), and EQ-5D visual analog scale (EQ-VAS). Postoperative overall satisfaction was evaluated using the following responses: “very satisfied,” “satisfied,” “unsatisfied,” or “very unsatisfied.” DAU was successful in 95.3% cases, with a median postoperative follow-up duration of 48 months. Multivariate logistic regression analysis revealed that “greater blood loss” was an independent predictor of failed urethroplasty. Questionnaire responses were obtained from 80.1% patients. The mean LUTS-related QOL, EQ-5D score and EQ-VAS improved significantly from 2.8, 0.63 and 54.4 at baseline to 0.9, 0.81 and 76.6 postoperatively (*p* < 0.0001 for all parameters). Moreover, 35.5% and 59.2% of the patients responded being “satisfied” and “very satisfied,” respectively, with their DAU outcomes. DAU not only had a high surgical success rate, but also a significant beneficial effect on both LUTS-related QOL and overall health-related QOL.

## 1. Introduction

Pelvic fracture urethral injury (PFUI) is a rare injury associated with pelvic fractures caused by blunt force [1,2]. Early studies estimated a PFUI incidence of 10–25% among men with pelvic fractures, with subsequent studies reporting a much lower incidence (1.4 ≈ 2%) [2,3,4]. PFUI is relatively rare and more common in males since the female urethra is shorter and more mobile than the male urethra; moreover, it is almost completely protected by the pubic bone [4,5]. Although PFUI itself is not lethal, it can cause urinary retention and sepsis due to urine extravasation in the acute phase. Further, it can cause significant morbidity, including urethral gap, erectile dysfunction (ED), and urinary incontinence (UI) [1]. The standard treatment for the post-PFUI urethral gap is delayed anastomotic urethroplasty (DAU) via a perineal approach [1,6]. DAU has a simple concept; specifically, complete removal of the trauma-induced scar and sequential use of the four ancillary techniques (bulbar urethral mobilization, corporal splitting, inferior partial pubectomy, and supracrural urethral rerouting) as required to achieve tension-free urethral anastomosis [7]. DAU by experienced surgeons has a fairly good success rate, which generally exceeds 90% [8,9,10,11,12,13,14,15]. Since 2004, our hospital has been a referral center for urethral reconstruction and is now the largest of its kind in Japan. In 2019, we reported the outcomes of 115 patients with DAU who visited our hospital within the past 10 years [12]. Since then, we have gained more experience and almost doubled the number of previously reported cases. This article presents an updated series of DAU for PFUI; moreover, we aimed to evaluate its effectiveness with respect to surgical and patient-reported outcomes.

## 2. Patients and Methods

### 2.1. Patient Selection and Evaluation of Urethral Gap

This study was approved by the Institutional Review Board of the National Defense Medical College (approval number 4154). We retrospectively reviewed 211 patients who underwent DAU at the National Defense Medical College Hospital or our affiliated institutions from January 2008 to June 2021. The urethral gap was evaluated at ≥3 months after pelvic trauma or after the last urethral manipulation for urethral rest [16]. Suprapubic tube (SPT) was performed in patients with decreased urinary stream or urinary retention due to interruption resulting from urethral manipulations, including direct vision internal urethrotomy (DVIU) or urethral dilatation. Urethral gap length, urethral injury site (i.e., either at the prostatic apex, within the membranous urethra, or at the bulbomembranous junction), and bladder neck competency were assessed through retrograde and antegrade urethrography, as well as antegrade flexible cystoscopy via SPT tracts. In principle, pelvic contrast-enhanced magnetic resonance imaging was performed to evaluate periurethral information and help select the repair type [17].

### 2.2. Surgical Technique

The disease duration was defined as the time from the day of injury to the DAU. DAUs were performed by a single surgeon (A.H.) with the stepwise use of ancillary techniques based on intraoperative findings [7]. Briefly, under general anesthesia, the patient was placed in a high lithotomy position; moreover, the bulbar urethra was circumferentially dissected through a midline perineal incision and fully mobilized distally up to the penoscrotal junction. Subsequently, the bulbar urethra was transected at the obstruction site; further, the location of the proximal urethral end was identified. The scar tissue covering the urethral end was meticulously excised to reach the normal urethral mucosa. A urethral anastomosis was performed using eight interrupted 4-0 PDS sutures, followed by placement of a 16Fr Foley catheter. In cases where the proximal urethral end could not be identified due to a thick scar or urethral deviation, or where tension-free anastomosis was impossible due to a long urethral gap, corporal splitting, inferior partial pubectomy, and supracrural urethral rerouting were performed in that order [7]. For patients at risk of rectal injury during transperineal manipulation due to rectal bulging in the disrupted urethral gap, or those with limited bulbar urethral length due to previously failed urethroplasty, a combined transperineal and transperitoneal approach was used. A simple perineal approach was defined as one that could be accomplished through only bulbar urethral mobilization or corporal splitting. Moreover, an elaborate approach was defined as one requiring further ancillary techniques [1]. A urethral catheter was placed for two weeks after DAU; moreover, pericatheter retrograde urethrography was performed to check the anastomosis healing. If the anastomosis was patent and there was no contrast medium leakage, the urethral catheter was removed and voiding cystourethrography was performed after clamping the SPT. If the patient could void without problems for several days, the SPT was removed. The severity of perioperative complications was categorized based on the Clavien–Dindo classification.

### 2.3. Postoperative Follow-Up

Patients were postoperatively followed-up at the National Defense Medical College Hospital for 3, 6, and 12 months, and subsequently annually, using questionnaires, uroflowmetry, measurement of the postvoid residual urine volume (PVR), and flexible cystoscopy. Patients who lived far away from the National Defense Medical College Hospital and had difficulty making regular visits were followed-up by a referring physician in their neighborhood. Surgical success was indicated by a sufficiently large urethral lumen that allowed a 17Fr flexible cystoscope to pass through the urethral anastomosis without resistance and the lack of required additional treatment. We used the following questionnaires: the validated urethral stricture surgery patient-reported outcome measure (USS-PROM), which was originally developed in the UK [18] and translated into Japanese [19]; the International Consultation on Incontinence-Questionnaire Short Form (ICIQ-SF) [20]; daily pad use for UI; and the Sexual Health Inventory for Men (SHIM). The USS-PROM consists of six questions regarding lower urinary tract symptoms (LUTS) (total scores of 0 and 24 indicating asymptomatic and most symptomatic, respectively), LUTS-specific quality of life (QOL) (score 0 = not at all, 1 = a little, 2 = somewhat, 3 = a lot), Peeling’s picture score [21], and health-related QOL using the EuroQol-5 dimensions (EQ-5D) index and EQ-5D visual analog scale (EQ-VAS) [22]. The overall postoperative patient satisfaction was assessed by asking the patients to select “very satisfied”, “satisfied”, “unsatisfied”, or “very unsatisfied” [18,19].

### 2.4. Statistical Analysis

All statistical analyses were performed using JMP software version 14 (SAS Institute Inc., Cary, NC, USA). Data are presented as the median and interquartile range (IQR) or mean values. The Wilcoxon rank-sum test was used to evaluate relationships between continuous data. Pearson’s chi-squared test was used to assess categorical data. Changes in QOL-related parameters were compared using the Wilcoxon signed-rank test. Univariate and multivariate logistic regression analyses were used to identify independent predictors of surgical success and postoperative satisfaction. Statistical significance was set at *p* < 0.05.

## 3. Results

### 3.1. Patient Characteristics

Table 1 shows the patients’ background characteristics. Based information in the referral letter, pelvic trauma was caused by traffic accidents, occupational accidents, and fall from height in 106 (50.2%), 96 (45.5%), and 9 (4.3%) patients, respectively. The median (IQR) age, preoperative body mass index, and disease duration were 42 (26–58) years, 22.3 (20.4–24.9) kg/m^2^, and 13 (10–26) months, respectively. The initial treatment provided by the previous physician was SPT placement and primary realignment in 146 (69.2%) and 65 (30.8%) patients, respectively. Before referral to our hospital, 69 (32.7%) patients had at least one previous failed transurethral treatment, including DVIU or urethral dilation; moreover, 25 (11.8%) patients had at least one previous failed urethroplasty for PFUI. The bladder neck was intact and open in 189 (89.6%) and 22 (10.4%) cases, respectively. The urethral injury site was the bulbomembranous junction, membranous urethra, and prostatic apex in 112 (58.1%), 75 (35.5%), and 24 (11.4%) cases, respectively. The median (IQR) urethral gap length was 14 (10–24) mm.

### 3.2. Surgical Outcome

The urethroplasty type was a simple perineal approach and an elaborate approach in 104 (49.3%) and 107 (50.7%) patients, respectively. Ten (4.7%) patients required a combined transabdominal approach. The required ancillary techniques were bulbar urethral mobilization alone in 7 (3.3%) patients, bulbar urethral mobilization and corporal splitting in 97 (46.0%) patients; bulbar mobilization, corporal splitting, and inferior partial pubectomy in 97 (46.0%); and bulbar mobilization, corporal splitting, partial pubectomy, and supracrural urethral rerouting in 10 (4.7%) patients. The median operative time and blood loss were 207 (176–238) minutes and 127 (60–240) mL, respectively. Postoperative complications included compartment syndrome of the lower extremity in one (0.5%, grade 1) patient, peroneal nerve palsy in two (1.0%, grade 1) patients, scrotal hematoma in three (1.4%, grade 1) patients, surgical site infection in one (0.5%, grade 1) patient, and periurethral abscess in two (1.0%; grade 2 in one and grade 3 in one) patients. Table 2 shows the differences in clinical backgrounds between patients who underwent a simple perineal approach and an elaborate approach.

At a median of 48 (1–80) postoperative months, DAU was successful in 201 (95.3%) patients. Table 3 shows the clinical backgrounds of the 10 patients categorized as having failed DAU. The median (IQR) time between DAU and failure was 3 (3–7) months. Six out of the ten patients could urinate after DAU; however, the urethral lumen was too narrow to allow passage of a 17Fr flexible cystoscope through the urethral anastomosis without resistance. The anastomotic stenosis in all six cases was short (<5 mm) and flimsy. Three patients were followed-up without additional treatment due to a lack of subjective symptoms, while three other patients underwent additional DVIU (with two achieving stenosis-free status). The remaining four patients could not void after DAU due to anastomotic stenosis. Among them, two had relatively long anastomotic stenosis (25 mm in one and 20 mm in one); however, they had adequate remaining bulbar urethra and could be salvaged by repeated DAU through a transpubic approach as described [23,24], and currently achieved a stenosis-free status. The remaining two patients showed bulbar urethral necrosis (BUN) [25,26] and continued to undergo SPT.

Table 4 shows the relationships between the surgical outcomes and clinical parameters. Patients with failed DAU had a significantly higher median intraoperative blood loss (235 mL) than patients with successful DAU (125 mL, *p* = 0.02). Multivariate logistic regression analysis revealed that high intraoperative blood loss was an independent predictor of urethroplasty failure (odds ratio [OR] 1.002, 95% confidence interval [CI] 1.00–1.01, *p* = 0.02; Table 5).

### 3.3. Patient-Reported Outcome

Patient-reported outcomes were assessed in 169 out of 211 patients (80.1%) within a median of 13 postoperative months (IQR 12–24) (Table 6), while 30 (14.2%) and 12 (5.7%) patients could not be assessed due to distant location and lack of cooperation/missing data, respectively. The median maximum urinary flow rate (Qmax) and PVR were 19.6 (13.5–25.7) mL/s and 20 (11–42) mL, respectively. The median LUTS score and Peeling’s picture score were 4 (1.5–7.5) and 2 (2–3), respectively. The median ICIQ-SF and number of pads used per day were 4 (0–9) and 0 (0–1), respectively; furthermore, 101 (59.8%) patients achieved pad-free status. Table 7 shows the distribution of the SHIM scores before and after the DAU. Very few patients (3 [1.8%] before DAU and 1 [0.6%] after DAU) experienced normal erectile function (SHIM 22–25 points), with most of the patients experiencing severe ED or no sexual activity at all. There was no significant difference between the mean preoperative and postoperative SHIM scores (3.8 vs. 4.2, 95% CI 0.5–1.3, *p* = 0.35).

Moreover, 60 (35.5%) and 100 (59.2%) patients were “satisfied” and “very satisfied” respectively, with the outcomes of DAU; moreover, nine (5.3%) patients were “unsatisfied”. Among the nine “unsatisfied” patients, two responded that “the urinary condition did not improve”, six responded that “the urinary condition improved but there was some other problem” and one responded that “the urinary condition did not improve and there was some other problem as well”. Table 8 presents the relationships between patient satisfaction and clinical parameters. Compared with the “satisfied” and “unsatisfied” patients, the “very satisfied” patients were younger (*p* = 0.009); had a lower percentage of preoperative urinary retention (*p* = 0.04); and had significantly lower postoperative LUTS scores (*p* = 0.0003), Peeling’s picture scores (*p* = 0.0002), and ICIQ-SF total scores (*p* = 0.001) (Table 8). Multivariate logistic regression analysis revealed that age (OR 0.91, 95% CI 0.94–0.99, *p* = 0.01), preoperative urinary retention (OR 0.21, 95% CI 0.06–0.84, *p* = 0.03), Peeling’s picture score (OR 0.49, 95% CI 0.27–0.89, *p* = 0.02), and SHIM score (OR 1.10, 95% CI 1.01–1.19, *p* = 0.01) were independent predictors of “very satisfied” responses (Table 9). Table 10 shows the postoperative changes in LUTS and health-related QOL. The mean LUTS-specific QOL score significantly improved from 2.8 at baseline to 0.9 after urethroplasty (mean difference = 1.9, <0.0001). The mean EQ-5D index score and EQ-VAS score significantly improved from 0.63 and 54.4, respectively, at baseline to 0.81 and 76.6, respectively, after urethroplasty (mean difference = 0.18 and 22.2, *p* < 0.0001 and *p* < 0.0001, respectively).

## 4. Discussion

DAU is considered the gold standard for PFUI in men and has become a familiar procedure among reconstructive urologists worldwide. However, DAU remains unfamiliar and difficult for most urologists. The difficulty of DAU results from the complexity of its procedure and its rarity. In developed countries, there has been a decrease in the number of patients with trauma due to improved working conditions and road safety, including airbags in automobiles [27,28,29]. A nationwide survey on surgical management of PFUI by certified urologists in Japan reported that 60% had surgically treated less than three patients in their entire career, while only 5% had treated >10 patients [28]. Furthermore, most of the treatments chosen were transurethral treatments, including DVIU and urethral dilation, which are currently considered inappropriate [28]. Transurethral treatments for PFUI are futile and unnecessarily prolong the time to cure the urethral gap; moreover, they involve risks complicating urethral gap due to iatrogenic urethral injury [30,31,32]. The DAU for PFUI is among the most challenging procedures in urology [1,33]. The surgeon must use various ancillary techniques, as required, to identify the disrupted urethral end and perform an accurate anastomosis in the small surgical field of the perineum [34]. Surgeons’ technical inexperience is the most common cause of urethroplasty failure [12,35]. Mundy recommends that urologists who do not routinely perform >15 urethroplasties per year should refer their patients to high-volume centers with sufficient caseloads for maintaining surgical expertise [36]. The best chance for curing the urethral gap is at the first attempt; additionally, the patient’s QOL is dependent on the surgeon’s decision [37]. Patients with a post-PFUI urethral gap should be referred to a specialist without performing futile DVIU or dilatation.

DAU seeks to achieve tension-free urethral anastomosis after adequate mobilization of the bulbar urethra and sequential use of ancillary techniques, including corporal splitting, inferior partial pubectomy, and urethral rerouting, as required [7]. Indications for assistive techniques are dependent on the severity of the PFUI and the surgeon’s preference. Some surgeons consider that little or no concomitant use of ancillary techniques other than bulbar urethral mobilization is necessary [9]; contrastingly, others frequently use further techniques [14,38,39]. Andrich et al. reported that 58 out of 100 patients required an elaborate approach since surgeons were inclined to proceed with the more familiar ancillary technique [38]. In addition, a review of 120 DAUs performed over a 10-year period conducted by Flynn et al. showed an increase in the number of patients choosing an elaborate approach, which was attributed to increased confidence in the safety and efficacy of ancillary techniques [14]. We previously applied an elaborate approach to 56.5% of patients [12]. Although this percentage decreased to 50.7% in the current series, it was still higher than that in other reports. We believe there are two reasons for this high frequency. First, the actual urethral gap length is longer than the length assessed by urethrography; that is, excision of the fibrotic tissues at the urethral ends leads to an increase in the urethral gap and elaborated preference. Second, the most difficult point in DAU is identifying the urethral end and accurate anastomosis in the narrow field of view from the pelvic perineum, where the anatomy has been disrupted by pelvic trauma. Partial pubectomy seeks to not only reduce anastomotic tension, but also ensure a good view and space for manipulation in the pelvis. Either way, reconstructive urologists should be familiar with all ancillary techniques and should handle them whenever necessary [38].

The definition of successful DAU remains unclear; furthermore, there is no consensus regarding the optimal postoperative follow-up strategy [8,9,10,11,13,14,15]. We adopted a strict definition of success based on cystoscopy-based anatomical findings. Ten (4.7%) patients were judged to have failed DAU, while three were asymptomatic and did not require additional treatment. Various clinical parameters, including the long urethral gap length, history of angioembolization, bulbar urethral status, lateral prostatic displacement, and incomplete scar excision, are associated with post-DAU anastomotic stenosis [11,40]. In our series, only excessive bleeding was associated with failure of urethroplasty, which is consistent with our previous findings [12]. In patients undergoing radical prostatectomy for prostate cancer, excessive bleeding is associated with the development of stenosis of the vesicourethral anastomosis, which may result from inadequate visualization and compromised anastomotic quality [41,42]. Accordingly, excessive bleeding may reduce the quality of the urethral anastomosis by obstructing the surgical field visibility and impeding delicate handling.

In addition to surgical outcomes, patient-reported outcomes are becoming increasingly important during follow-up after urethroplasty. We used the Japanese translation of the USS-PROM for the follow-up assessment of DAU [19]. To assess ED and UI in patients with PFUI, we used the SHIM and ICIQ-SF together, which were not included in the original English version of the USS-PROM. ED frequently occurs after PFUI; additionally, since most patients are young and have a long life expectancy, proper management can greatly improve the QOL. A recent meta-analysis estimated that the average incidence of ED in patients with PFUI was 34% [43,44]. Compared with the reported series, our cohort had a markedly higher proportion of patients with ED. There was no significant post-intervention difference in SHIM scores, which is consistent with a previous report that DAU is unlikely to be a direct cause of ED; rather, it may result from disruption of the neurovascular structure caused by pelvic trauma [14,45]. Koraitim reported that spontaneous recovery of sexual function can occur up to two years after injury; further, the preferred time for assessing sexual function is two years after injury [45]. Since the SHIM score was evaluated at a median of 13 postoperative months, it may be necessary to re-evaluate sexual function.

Pelvic trauma can damage two components of male urinary continence; specifically, the internal (bladder neck) and external (membranous urethra) sphincters, and UI can occur after DAU. Urinary continence in patients with PFUI was thought to be dependent on the bladder neck function since the urethra is disrupted at the prostatic apex and the external sphincter function is lost [46,47]. However, it was subsequently found that urethral injury most often occurs at the bulbomembranous junction, with the external sphincter function being often preserved to some extent [48,49,50]. The incidence of UI in patients with PFUI ranges from 2% to 25%, which widely varies across reports, possibly due to the inconsistent definitions of UI [13,15,51,52,53,54]. In our cohort, 101 (59.8%) patients achieved a pad-free status. Although the ICIQ-SF score was not a predictor of “very satisfied” patients in the multivariate analysis, urinary continence can affect both the LUTS-specific QOL and health-related QOL. Excessive dissection of the remaining membranous urethra and excessive use of energy devices should be avoided to preserve urinary continence.

The most significant limitation of this study is that we could not assess the patient-reported outcomes for all included patients. Many of our included patients lived far from our institute, which impeded them from making regular postoperative visits. Moreover, since referring physicians were following up on these patients, we could not capture all the data. Nonetheless, this was a large series that evaluated both surgical and patient-reported outcomes of DAU for PFUI and could provide useful information for PFUI management by reconstructive urologists.

## 5. Conclusions

DAU for PFUI has a high surgical success rate and a significant beneficial effect on both LUTS-specific and health-related QOL. Careful manipulation within a bloodless operative field is key to successful DAU.

## Figures and Tables

**Table 1:** Patient characteristics.

Number of patients	211
Age (years), median (IQR)	42 (26–58)
BMI (kg/m^2^), median (IQR)	22.3 (20.4–24.9)
Disease duration (months), median (IQR)	13 (10–26)
Current smoking, *n* (%)	62 (29.4)
History of COPD, *n* (%)	8 (3.8)
History of DM, *n* (%)	11 (5.2)
History of IHD, *n* (%)	4 (1.9)
Urinary retention, *n* (%)	187 (88.6)
Initial management (%)	
SPT	146 (69.2)
PR	65 (30.8)
DVIU and/or dilation history prior to referral, *n* (%)	69 (32.7)
Urethroplasty history prior to referral, *n* (%)	25 (11.8)
Bladder neck status on cystoscopy and/or cystourethrogram (%)	
Intact	189 (89.6)
Open	22 (10.4)
Urethral injury site, *n* (%)	
Prostatic apex	24 (11.4)
Membranous/bulbomembranous urethra	187 (88.6)
Urethral gap length (mm), median (IQR)	14 (10–24)
Simple perineal/Elaborate approach (%)	
Simple	104 (49.3)
Elaborate	107 (50.7)
Operative time (minutes), median (IQR)	207 (176–238)
Blood loss (mL), median (IQR)	127 (60–240)
Urethroplasty success (%) * CS-based	201 (95.3)
Postoperative periods (months), median (IQR)	48 (31–80)

* A sufficiently large urethral lumen that allowed a 17Fr flexible cystoscope to pass through the urethral anastomosis without resistance. BMI, body mass index; COPD, chronic obstructive pulmonary disease; DM, diabetes mellitus; DVIU, direct vision internal urethrotomy; IHD, ischemic heart disease; IQR, interquartile range; PR, primary realignment, SPT: suprapubic tube.

**Table 2:** The association of delayed anastomotic urethroplasty (DAU) type with clinical parameters.

		Simple Perineal	Elaborate	*p*
Patients (%)				
Age, median (IQR)		45 (27–60)	40 (25–58)	0.19
BMI (kg/m^2^), median (IQR)		22.6 (20.5–25.1)	21.8 (20.0–24.8)	0.27
Initial management, *n* (%)				
	SPT	64 (43.8)	82 (56.2)	0.02
	PR	40 (61.5)	25 (38.5)	
Gap length on urethrogram (mm), median (IQR)		12 (8–15)	18 (13–29)	<0.0001
Bladder neck status on cystoscopy and/or cystourethrogram (%)				
	Intact	94 (49.7)	95 (50.3)	0.7
	Open	10 (45.5)	12 (54.5)	
Site of urethral injury, *n* (%)				
	Prostatic apex	10 (41.7)	14 (58.3)	0.43
	Membranous/bulbomembranous urethra	94 (50.3)	93 (49.7)	
History of DVIU/dilation, *n* (%)		27 (25.9)	42 (39.3)	0.04
Salvage urethroplasty, *n* (%)		7 (6.7)	18 (16.8)	0.02
Operative time (mins), median (IQR)		187 (163–216)	226 (199–286)	<0.0001
Blood loss (mL), median (IQR)		86 (43–184)	168 (88–295)	<0.0001

BMI, body mass index; DVIU, direct vision internal urethrotomy; IQR, interquartile range; PR, primary realignment; SPT, suprapubic tube.

**Table 3:** Clinical characteristics of patients with failed DAUs.

Case	Age (Years)	Primary/Redo	Time to Failure (Months)	Status of the Remaining Urethral Gap	Status of the Remaining Bulbar Urethra	Additional Treatment (Consequence)
1	38	Primary	3	<5 mm, flimsy	Normal	Watchful waiting
2	19	Primary	8	<5 mm, flimsy	Normal	Watchful waiting
3	21	Primary	6	<5 mm, flimsy	Normal	Watchful waiting
4	23	Primary	5	<5 mm, flimsy	Normal	DVIU × 1 (successful)
5	69	Primary	3	<5 mm, flimsy	Normal	DVIU × 1 (successful)
6	58	Primary	10	<5 mm, flimsy	Normal	DVIU × 1 (failed)
7	17	Primary	4	25 mm, dense	Normal	Redo DAU (successful)
8	55	Primary	3	20 mm, dense	Normal	Redo DAU (successful)
9	69	Primary	4	35 mm, dense	BUN	Chronic SPT placement
10	65	Redo	1	30 mm, dense	BUN	Chronic SPT placement

BUN, bulbar urethral necrosis; DAU, delayed anastomotic urethroplasty; DVIU, direct vision internal urethrotomy; SPT, suprapubic tube.

**Table 4:** The association of surgical outcomes with clinical parameters.

		Successful	Failed	*p*
Patients (%)		201 (95.3)	10 (4.7)	
Age, median (IQR)		42 (27–58)	47 (21–66)	0.87
BMI (kg/m^2^), median (IQR)		22.4 (20.2–25.1)	21.5 (20.9–24.3)	0.86
Disease duration (month), median (IQR)		13 (10–26)	11 (6–572)	0.54
Current smoking, *n* (%)		58 (28.9)	4 (40.0)	0.45
History of COPD, *n* (%)		8 (3.9)	0 (0.0)	0.52
History of DM, *n* (%)		11 (5.5)	0 (0.0)	0.48
History of IHD, *n* (%)		3 (1.5)	1 (10.0)	0.05
Retention, *n* (%)		179 (89.1)	8 (80.0)	0.38
Initial management, *n* (%)				
	SPT	140 (95.9)	6 (4.1)	0.52
	PR	61 (93.8)	4 (6.2)	
Gap length on urethrogram (mm), median (IQR)		14 (10–23)	12 (7–21)	0.35
Bladder neck status (%)				
	Intact	180 (95.2)	9 (4.8)	0.96
	Open	21 (95.5)	1 (4.5)	
Site of urehtral injury, *n* (%)				
	Prostatic apex	21 (87.5)	3 (12.5)	0.06
	Membranous/bulbomembranous urethra	180 (96.3)	7 (3.7)	
History of DVIU/dilation, *n* (%)		64 (31.8)	5 (50.0)	0.23
Salvage urethroplasty, *n* (%)		24 (11.9)	1 (10.0)	0.85
Simple/Elaborate, *n* (%)				
	Simple	101 (97.1)	3 (2.9)	0.21
	Elaborate	100 (93.5)	7 (6.5)	
Operative time (mins), median (IQR)		207 (176–235)	226 (183–303)	0.37
Blood loss (mL), median (IQR)		125 (59–234)	235 (134–862)	0.02

BMI, body mass index; COPD, chronic obstructive pulmonary disease; DM, diabetes mellitus; DVIU, direct vision internal urethrotomy; IHD, ischemic heart disease; IQR, interquartile range; PR, primary realignment; SPT, suprapubic tube.

**Table 5:** Logistic regression analysis for predictors of failed urethroplasty.

	Univariate			Multivariate		
	OR	95% CI	*p*	OR	95% CI	*p*
Age (year)	1.001	0.97–1.04	0.94			
BMI (kg/m^2^)	0.97	0.80–1.17	0.75			
Current smoking (yes)	1.64	0.45–6.04	0.46			
IHD history (yes)	7.30	0.69–77.64	0.16			
Preoperative urinary retention (yes)	0.49	0.10–2.46	0.42			
Initial management (PR)	1.53	0.42–5.62	0.53			
Prior DVIU/dilation (yes)	2.14	0.59–7.66	0.25			
Prior urethroplasty (yes)	0.82	0.10–6.76	0.85			
Urethral gap length (mm)	0.97	0.89–1.04	0.37			
Bladder neck status (open)	0.95	0.11–7.89	0.96			
Site of urethral injury (prostatic apex)	3.67	0.88–15.29	0.10			
Type of urethroplasty (elaborate)	2.36	0.59–9.37	0.20			
Operative time (minute)	1.004	0.995–1.013	0.39			
Blood loss (mL)	1.003	1.001–1.004	0.004	1.002	1.000–1.005	0.02

BMI, body mass index; CI, confidence interval; DVIU, direct vision internal urethrotomy; IHD, ischemic heart disease; PR, primary realignment.

**Table 6:** Post-DAU clinical parameters including patient-reported outcomes.

Number of patients	169
Qmax (mL/s), median (IQR)	19.6 (13.5–25.7)
PVR (mL), median (IQR)	20 (11–42)
LUTS-score, median (IQR)	4 (1.5–7.5)
Peeling’s score, median (IQR)	2 (2–3)
ICIQ-SF, median (IQR)	4 (0–9)
Daily pad use, median (IQR)	0 (0–1)
Satisfaction, *n* (%)	
Very satisfied	100 (59.2)
Satisfied	60 (35.5)
Unsatisfied	9 (5.3)
Very unsatisfied	0 (0.0)

DAU, delayed anastomotic urethroplasty; ICIQ-SF, International Consultation on Incontinence-Questionnaire Short Form; IQR, interquartile range; LUTS, lower urinary tract symptoms; PVR, post-void residual urine volume; Qmax, maximum flow rate.

**Table 7:** Distribution of ED.

Severity of ED (Total SHIM Score)	Pre-DAU (%)	Post-DAU (%)
Normal (22–25)	3 (1.8)	1 (0.6)
Mild (17–21)	6 (3.6)	13 (7.7)
Mild to Moderate (12–16)	7 (4.1)	9 (5.3)
Moderate (8–11)	7 (4.1)	9 (5.3)
Severe (5–7)	25 (14.8)	24 (14.2)
Unavailable (0–4)	121 (71.6)	113 (66.9)

ED, erectile dysfunction; SHIM, Sexual Health Inventory for Men; DAU, delayed anastomotic urethroplasty.

**Table 8:** Association between satisfaction and parameters.

Parameter	Very Satisfied	Satisfied/Unsatisfied	*p*
Number of patients	100	60/9	
Age (median, IQR)	41 (23–56)	47 (35–63)	0.009
BMI (median, IQR)	22.6 (20.4–25.4)	22.3 (20.5–24.7)	0.94
Disease duration (months, median, IQR)	14 (10–29)	14 (11–30)	0.96
Urinary retention, *n* (%)	84 (84.0)	65 (94.2)	0.04
DVIU/dilation history, *n* (%)	32 (32.0)	28 (40.6)	0.25
Prior urethroplasty, *n* (%)	12 (12.0)	9 (13.0)	0.84
Surgical suceess, *n* (%)	96 (96.0)	67 (97.1)	0.7
Qmax, (mL/s, median, IQR)	19.9 (14.4–26.2)	18.9 (12.3–23.8)	0.34
PVR (mL. median, IQR)	19 (11–39)	24 (11–46)	0.25
LUTS-score (median, IQR)	3 (1–6)	6 (3–10)	0.0003
Peeling score (median, IQR)	2 (2–3)	3 (2–3)	0.0002
post SHIM (median, IQR)	2 (0–5)	2 (0–5)	0.85
ICIQ-SF score (median, IQR)	4 (0–8)	7 (3–11)	0.001

BMI, body mass index; DVIU, direct vision internal urethrotomy; ICIQ-SF, International Consultation on Incontinence-Questionnaire Short Form; IQR, interquartile range; LUTS, lower urinary tract symptoms; PVR, post-void residual urine volume; Qmax, maximum flow rate; SHIM, Sexual Health Inventory for Men.

**Table 9:** Logistic regresssion analysis of predicting “very satisfied” patients.

	Univariate			Multivariate		
	OR	95% CI	*p*	OR	95% CI	*p*
Age (year)	0.98	0.95–0.99	0.009	0.97	0.94–0.99	0.01
BMI (kg/m^2^)	1.01	0.92–1.09	0.88			
Disease duration (month)	0.99	0.99–1.00	0.86			
Urinary retention (yes)	0.32	0.10–1.01	0.04	0.21	0.06–0.84	0.03
DVIU/dilation history (yes)	0.69	0.36–1.30	0.25			
Prior urethroplasty (yes)	0.91	0.36–2.29	0.84			
Surgical success (yes)	1.39	0.25–7.85	0.7			
Qmax (mL/s)	1.01	0.98–1.05	0.48			
PVR (mL)	0.99	0.99–1.01	0.59			
LUTS-score	0.87	0.80–0.94	0.0001			
Peeling score	0.47	0.31–0.71	0.0002	0.49	0.27–0.89	0.02
post SHIM	1.01	0.96–1.07	0.59	1.10	1.01–1.19	0.01
ICIQ-SF score	0.9	0.85–0.96	0.0007			

BMI, body mass index; DVIU, direct vision internal urethrotomy; ICIQ-SF, International Consultation on Incontinence-Questionnaire Short Form; LUTS, lower urinary tract symptoms; PVR, post-void residual urine volume; Qmax, maximum flow rate; SHIM, Sexual Health Inventory for Men; OR, odds ratio; CI, confidence interval.

**Table 10:** Comparison of preoperative and postoperative parameters.

	Preoperative Mean	Postoperative Mean	*p*	Mean of Differences	95% CI of Mean of Differences
LUTS-QOL	2.8	0.9	<0.0001	1.9	1.8–2.1
EQ-VAS	54.4	76.6	<0.0001	22.2	18.8–25.6
EQ-5D score	0.63	0.81	<0.0001	0.18	0.15–0.21

CI, confidence interval; EQ-5D, EuroQol-5 dimensions; EQ-VAS, EuroQol-5 dimensions visual analogue scale; LUTS, lower urinary tract symptoms; QOL, quality of life.

## Data Availability

The data presented in this study are available upon request from the corresponding author.

## References

[1] Gomez R.G., Mundy T., Dubey D., El-Kassaby A.W., Firdaoessaleh, Kodama R., Santucci R. (2014). SIU/ICUD consultation on urethral strictures: Pelvic fracture urethral injuries. Urology.

[2] Bjurlin M.A., Fantus R.J., Mellett M.M., Goble S.M. (2009). Genitourinary injuries in pelvic fracture morbidity and mortality using the National Trauma Data Bank. J. Trauma.

[3] Bhatt N.R., Merchant R., Davis N.F., Leonard M., O’Daly B.J., Manecksha R.P., Quinlan J.F. (2018). Incidence and immediate management of genitourinary injuries in pelvic and acetabular trauma: A 10-year retrospective study. BJU Int..

[4] Johnsen N.V., Dmochowski R.R., Young J.B., Guillamondegui O.D. (2017). Epidemiology of Blunt Lower Urinary Tract Trauma with and Without Pelvic Fracture. Urology.

[5] Hagedorn J.C., Voelzke B.B. (2015). Pelvic-fracture urethral injury in children. Arab. J. Urol..

[6] Morey A.F., Broghammer J.A., Hollowell C.M.P., McKibben M.J., Souter L. (2021). Urotrauma Guideline 2020: AUA Guideline. J. Urol..

[7] Webster G.D., Ramon J. (1991). Repair of pelvic fracture posterior urethral defects using an elaborated perineal approach: Experience with 74 cases. J Urol..

[8] Koraitim M.M. (2005). On the art of anastomotic posterior urethroplasty: A 27-year experience. J. Urol..

[9] Kizer W.S., Armenakas N.A., Brandes S.B., Cavalcanti A.G., Santucci R.A., Morey A.F. (2007). Simplified reconstruction of posterior urethral disruption defects: Limited role of supracrural rerouting. J. Urol..

[10] Joshi P.M., Kulkarni S.B. (2020). Management of pelvic fracture urethral injuries in the developing world. World J. Urol..

[11] Johnsen N.V., Moses R.A., Elliott S.P., Vanni A.J., Baradaran N., Greear G., Smith T.G., Granieri M.A., Alsikafi N.F., Erickson B.A. (2020). Multicenter analysis of posterior urethroplasty complexity and outcomes following pelvic fracture urethral injury. World J. Urol..

[12] Horiguchi A., Shinchi M., Ojima K., Masunaga A., Ito K., Asano T., Takahashi E., Kimura F., Azuma R. (2019). Single-surgeon series of delayed anastomotic urethroplasty for pelvic fracture urethral injury: An analysis of surgical and patient-reported outcomes of a 10-year experience in a Japanese referral center. World J. Urol..

[13] Fu Q., Zhang J., Sa Y.L., Jin S.B., Xu Y.M. (2013). Recurrence and complications after transperineal bulboprostatic anastomosis for posterior urethral strictures resulting from pelvic fracture: A retrospective study from a urethral referral centre. BJU Int..

[14] Flynn B.J., Delvecchio F.C., Webster G.D. (2003). Perineal repair of pelvic fracture urethral distraction defects: Experience in 120 patients during the last 10 years. J. Urol..

[15] Cooperberg M.R., McAninch J.W., Alsikafi N.F., Elliott S.P. (2007). Urethral reconstruction for traumatic posterior urethral disruption: Outcomes of a 25-year experience. J. Urol..

[16] Terlecki R.P., Steele M.C., Valadez C., Morey A.F. (2011). Urethral rest: Role and rationale in preparation for anterior urethroplasty. Urology.

[17] Horiguchi A., Edo H., Soga S., Shinchi M., Masunaga A., Ito K., Asano T., Shinmoto H., Azuma R. (2018). Pubourethral Stump Angle Measured on Preoperative Magnetic Resonance Imaging Predicts Urethroplasty Type for Pelvic Fracture Urethral Injury Repair. Urology.

[18] Jackson M.J., Sciberras J., Mangera A., Brett A., Watkin N., N’Dow J.M.O., Chapple C.R., Andrich D.E., Pickard R.S., Mundy A.R. (2011). Defining a patient-reported outcome measure for urethral stricture surgery. Eur. Urol..

[19] Horiguchi A., Shinchi M., Ojima K., Masunaga A., Ito K., Asano T., Takahashi E., Kimura F., Azuma R. (2019). Evaluation of the effect of urethroplasty for anterior urethral strictures by a validated disease-specific patient-reported outcome measure. World J. Urol..

[20] Avery K., Donovan J., Peters T.J., Shaw C., Gotoh M., Abrams P. (2004). ICIQ: A brief and robust measure for evaluating the symptoms and impact of urinary incontinence. Neurourol. Urodyn..

[21] Peeling W.B. (1989). Diagnostic assessment of benign prostatic hyperplasia. Prostate Suppl..

[22] EuroQol G. (1990). EuroQol--a new facility for the measurement of health-related quality of life. Health Policy.

[23] Gupta N.P., Mishra S., Dogra P.N., Yadav R., Seth A., Kumar R. (2009). Transpubic urethroplasty for complex posterior urethral strictures: A single center experience. Urol. Int..

[24] Pratap A., Agrawal C., Tiwari A., Bhattarai B., Pandit R., Anchal N. (2006). Complex Posterior Urethral Disruptions: Management by Combined Abdominal Transpubic Perineal Urethroplasty. J. Urol..

[25] Kulkarni S.B., Orabi H., Kavanagh A., Joshi P.M. (2020). RE Re Do urethroplasty after multiple failed surgeries of pelvic fracture urethral injury. World J. Urol..

[26] Kulkarni S.B., Surana S., Desai D.J., Orabi H., Iyer S., Kulkarni J., Dumawat A., Joshi P.M. (2018). Management of complex and redo cases of pelvic fracture urethral injuries. Asian J. Urol..

[27] Kulkarni S.B., Barbagli G., Kulkarni J.S., Romano G., Lazzeri M. (2010). Posterior Urethral Stricture After Pelvic Fracture Urethral Distraction Defects in Developing and Developed Countries, and Choice of Surgical Technique. J. Urol..

[28] Kitahara S., Sato R., Yasuda K., Arai G., Nakai H., Okada H. (2008). Surgical treatment of urethral distraction defect associated with pelvic fracture: A nationwide survey in Japan. Int. J. Urol..

[29] Andrich D.E., Greenwell T.J., Mundy A.R. (2005). Treatment of pelvic fracture-related urethral trauma: A survey of current practice in the UK. BJU Int..

[30] Tausch T.J., Morey A.F., Scott J.F., Simhan J. (2014). Unintended negative consequences of primary endoscopic realignment for men with pelvic fracture urethral injuries. J. Urol..

[31] Johnsen N.V., Dmochowski R.R., Mock S., Reynolds W.S., Milam D.F., Kaufman M.R. (2015). Primary endoscopic realignment of urethral disruption injuries: A double-edged sword?. J. Urol..

[32] Horiguchi A., Shinchi M., Masunaga A., Okubo K., Kawamura K., Ojima K., Ito K., Asano T., Azuma R. (2017). Primary Realignment for Pelvic Fracture Urethral Injury Is Associated with Prolonged Time to Urethroplasty and Increased Stenosis Complexity. Urology.

[33] Pratap A., Agrawal C., Pandit R., Sapkota G., Anchal N. (2006). Factors Contributing to a Successful Outcome of Combined Abdominal Transpubic Perineal Urethroplasty for Complex Posterior Urethral Disruptions. J. Urol..

[34] Mundy A.R., Andrich D.E. (2011). Urethral trauma. Part II: Types of injury and their management. BJU Int..

[35] Gelman J. (2013). Tips for successful open surgical reconstruction of posterior urethral disruption injuries. Urol. Clin. N. Am..

[36] Mundy A.R. (2009). Words of wisdom. Re: Outcome of dorsal buccal graft urethroplasty for recurrent urethral strictures. Eur. Urol..

[37] Kulkarni S.B., Joshi P.M., Hunter C., Surana S., Shahrour W., Alhajeri F. (2015). Complex posterior urethral injury. Arab. J. Urol..

[38] Andrich D.E., O’Malley K.J., Summerton D.J., Greenwell T.J., Mundy A.R. (2003). The type of urethroplasty for a pelvic fracture urethral distraction defect cannot be predicted preoperatively. J. Urol..

[39] Joshi P.M., Batra V., Kulkarni S.B. (2019). Controversies in the management of pelvic fracture urethral distraction defects. Turk. J. Urol..

[40] Koraitim M.M., Kamel M.I. (2015). Perineal repair of pelvic fracture urethral injury: In pursuit of a successful outcome. BJU Int..

[41] Kostakopoulos A., Argiropoulos V., Protogerou V., Tekerlekis P., Melekos M. (2004). Vesicourethral anastomotic strictures after radical retropubic prostatectomy: The experience of a single institution. Urol. Int..

[42] Huang G., Lepor H. (2006). Factors predisposing to the development of anastomotic strictures in a single-surgeon series of radical retropubic prostatectomies. BJU Int..

[43] Chung P.H., Gehring C., Firoozabadi R., Voelzke B.B. (2018). Risk Stratification for Erectile Dysfunction After Pelvic Fracture Urethral Injuries. Urology.

[44] Blaschko S.D., Sanford M.T., Schlomer B.J., Alwaal A., Yang G., Villalta J.D., Wessells H., McAninch J.W., Breyer B.N. (2015). The incidence of erectile dysfunction after pelvic fracture urethral injury: A systematic review and meta-analysis. Arab. J. Urol..

[45] Koraitim M.M. (2013). Predictors of erectile dysfunction post pelvic fracture urethral injuries: A multivariate analysis. Urology.

[46] Pokorny M., Pontes J.E., Pierce J.M. (1979). Urological injuries associated with pelvic trauma. J. Urol..

[47] Colapinto V., McCallum R.W. (1977). Injury to the male posterior urethra in fractured pelvis: A new classification. J. Urol..

[48] Whitson J., McAninch J., Tanagho E., Metro M., Rahman N. (2008). Mechanism of Continence after Repair of Posterior Urethral Disruption: Evidence of Rhabdosphincter Activity. J. Urol..

[49] Mouraviev V.B., Santucci R.A. (2005). Cadaveric anatomy of pelvic fracture urethral distraction injury: Most injuries are distal to the external urinary sphincter. J. Urol..

[50] Andrich D.E., Mundy A.R. (2001). The nature of urethral injury in cases of pelvic fracture urethral trauma. J. Urol..

[51] Onen A., Ozturk H., Kaya M., Otcu S. (2005). Long-term outcome of posterior urethral rupture in boys: A comparison of different surgical modalities. Urology.

[52] Mouraviev V.B., Coburn M., Santucci R.A. (2005). The treatment of posterior urethral disruption associated with pelvic fractures: Comparative experience of early realignment versus delayed urethroplasty. J. Urol..

[53] Lumen N., Hoebeke P., Troyer B.D., Ysebaert B., Oosterlinck W. (2009). Perineal Anastomotic Urethroplasty for Posttraumatic Urethral Stricture with or Without Previous Urethral Manipulations: A Review of 61 Cases with Long-Term Followup. J. Urol..

[54] Corriere J.N. (2001). 1-Stage delayed bulboprostatic anastomotic repair of posterior urethral rupture: 60 patients with 1-year followup. J. Urol..

